# Prediabetes ¿de qué estamos hablando?

**DOI:** 10.1515/almed-2021-0030

**Published:** 2021-05-28

**Authors:** Javier Escalada San Martín

**Affiliations:** Departamento de Endocrinología y Nutrición, Clínica Universidad de Navarra, Pamplona, España; Sociedad Española de Endocrinología y Nutrición, Centro de Investigación Biomédica en Red de la Fisiopatología de la Obesidad y Nutrición (CIBEROBN), Madrid, España; Instituto de Investigación Sanitaria de Navarra (IdiSNA), Avenida Pío XII, 36, 31008, Pamplona, España

Las hiperglucemias intermedias (o estados prediabéticos) se refieren a dos entidades, la glucemia basal alterada (GBA) y la intolerancia a la glucosa (ITG), que se solapan y cuya definición ha variado en los últimos años, dependiendo de los niveles elegidos para definir la normoglucemia. Tanto la *American Diabetes Association* (ADA) como la *Organización Mundial de la Salud* (OMS) y la *Federación Internacional de Diabetes* (FID) establecen una categoría de estadios hiperglucémicos entre la normalidad glucémica y el diagnóstico de diabetes mellitus (DM) por la determinación de la glucemia basal plasmática o la glucemia en el plasma venoso tras SOG (sobrecarga oral de glucosa) de 75 g a las dos horas [1]. Más recientemente, se ha incorporado la posibilidad del diagnóstico en función de la concentración plasmática de HbA_1c_. Estas organizaciones difieren en la cifra de glucemia basal plasmática a partir de la que se considera GBA (100 vs. 110 mg/dL) y en la de la normalidad de HbA_1c_ (5,7% vs. 6%). En el momento actual, los criterios diagnósticos de prediabetes están bien establecidos y se muestran en la Figura 1.

**Figura 1:**
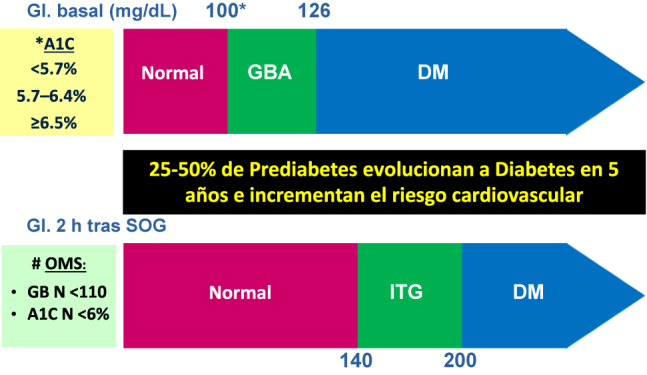
Diagnóstico de preDM y DM (ADA^*^ vs. OMS^#^). ADA, American Diabetes Association; DM, diabetes mellitus; GBA, glucemia basal alterada; Gl, glucemia; ITG, intolerancia a la glucosa; N, normal; OMS, Organización Mundial de la Salud; SOG, sobrecarga oral de glucosa

## ¿Por qué es importante el diagnóstico de estas entidades?

La prevalencia de prediabetes no es un tema menor. En España, la prevalencia de GBA es del 3,4% según los últimos datos publicados del estudio *di@bet.es* [2], y en el caso de la ITG su prevalencia es del 9,2%. Además, son situaciones con alto riesgo de desarrollo de diabetes y de enfermedad cardiovascular (CV). Así, los pacientes con GBA tienen un riesgo 5 veces superior de desarrollar DM [3] y su riesgo cardiovascular (infarto agudo de miocardio [IAM], accidentes cerebrovasculares [ACV] no fatales) es superior (riesgo relativo [RR]: 1,19), y también es superior la mortalidad (RR: 1,28) en comparación con la población sin diabetes [3]. Por su parte, la ITG está asociada con un mayor riesgo que la GBA de desarrollar diabetes. Este riesgo es 6 veces mayor que en los normoglucémicos (RR: 6,02 [IC 95%: 4,66 a 7,38]), y hasta 12 veces mayor en caso de asociarse ambas (RR: 12,21 [IC 95%: 4,32 a 20,10]) [3]. La ITG también implica un mayor riesgo de mortalidad cardiovascular (RR: 1,48) y general (RR: 1,66) [3].

Sin embargo, toda esta sólida argumentación se ha vista tambaleada en un estudio de cohortes recientemente publicado [4], basado en una comunidad de adultos mayores (edad media 75,6 años), en el que a pesar de una alta prevalencia de prediabetes (diagnóstico por GBA y/o HbA_1c_), la regresión a la normoglucemia o la muerte fueron más frecuentes que la progresión a diabetes, sugiriendo que la prediabetes puede no ser una entidad diagnóstica sólida en la vejez. Habrá que ver qué nos dicen otros estudios en esta población.

## ¿Qué podemos hacer?

Diversos estudios han analizado la eficacia de intervenciones farmacológicas y no farmacológicas en la prevención de diabetes y morbimortalidad cardiovascular en los estados prediabéticos [5], [6], [7], [8], [9], [10], [11], [12], [13], [14]. La efectividad de las intervenciones dirigidas a la prevención primaria de la DM2 se ha demostrado en pacientes con ITG, no en pacientes con GBA aislada o en diagnósticos de prediabetes basados en determinados valores de HbA_1c_.

Los estilos de vida y determinados fármacos antidiabéticos son eficaces en la prevención de la DM. Analizaremos a continuación algunos de los últimos estudios publicados sobre modificación de estilos de vida y sobre intervención farmacológica.

Uno de los últimos estudios publicados sobre modificación del estilo de vida es el *European Diabetes Intervention Study* [15], que es una continuación del estudio finlandés publicado en 2001 [9] y que se ha realizado en tres países europeos (Finlandia, Reino Unido y Países Bajos) para intentar confirmar los hallazgos del estudio inicial finlandés. Pues bien, tras un seguimiento de 3,1 años, la incidencia de DM fue un 57% menor en el grupo de intervención en comparación con el grupo control *(hazard ratio* [HR]: 0,42 [IC 95%: 0,29–0,60], p < 0,001). Los participantes que consiguieron una pérdida ponderal ≥ 5% al cabo de un año tuvieron una incidencia de DM un 65% menor (HR: 0,35 [IC 95%: 0,22–0,56], p < 0,001) y el mantenimiento de una pérdida de peso ≥ 5% durante dos y tres años se tradujo en una mayor reducción de la incidencia de diabetes (79% y 89% de reducción, respectivamente) [15]. Merece la pena mencionar los resultados de un estudio de intervención sobre estilos de vida en nuestro país, procedente del grupo DE-PLAN-CAT, en el que tras 4 años de seguimiento medio se consiguió una reducción en la incidencia de diabetes del 36,5% [16].

En cuanto a las intervenciones farmacológicas dirigidas a prevenir el desarrollo de diabetes, la mayor reducción en conversión a diabetes se ha obtenido en el estudio ACT-NOW, con pioglitazona [17], en el que se consiguió una reducción del 72% en el grupo tratado con pioglitazona respecto al placebo, aunque se acompañó de una ganancia ponderal de 3,9 kg frente a 0,8 kg (p < 0,001) y de un aumento de la incidencia de edema periférico (12,9% frente al 6,4%; p = 0,007). El estudio *Diabetes Prevention Program* (DPP) ya había demostrado años atrás que también metformina (850 mg dos veces al día) era eficaz en la reducción en conversión de prediabetes (ITG) a DM, en concreto en un 31% en comparación con placebo, aunque en ese estudio la intervención con medidas de estilo de vida consiguió una reducción de conversión a DM del 51% [10]. Finalmente, liraglutida (agonista del receptor de GLP-1) también ha demostrado reducir de manera importante la conversión a DM2 (2% en el grupo de tratamiento vs. 6% en el grupo control) en un estudio de 3 años de seguimiento [18].

En pacientes con obesidad mórbida, la estrategia más efectiva es la cirugía bariátrica (*odds ratio* 0,16 [IC 95%: 0,11–0,24]) [17].

El efecto sobre la morbimortalidad cardiovascular no ha podido demostrarse de forma concluyente debido a la corta duración de los estudios. La acarbosa se mostró eficaz en la disminución de las complicaciones cardiovasculares en el estudio STOP-NIDDM [12], pero este hallazgo se basa solo en 48 eventos y debe ser interpretado con mucha cautela, ya que el objetivo del estudio no era el efecto sobre la morbimortalidad cardiovascular.

En un metaanálisis recientemente publicado se demuestra que, en pacientes adultos con prediabetes, la utilización de medidas de estilo de vida (39%) o medicación (36%) es capaz de reducir la incidencia de DM2. Sin embargo, la medicación es menos eficaz a largo plazo (no se mantiene en el tiempo), mientras que la eficacia de la modificación del estilo de vida es capaz de prolongarse más allá de la propia intervención [19].

Por otro lado, en ese mismo análisis se demuestra lo conocido, es decir que la pérdida de peso es el factor esencial asociado a la progresión a DM2. Así, muestran que por cada kg de peso que se pierda se asocia a un 7% de reducción del riesgo de progresión a DM2, aspecto muy importante desde el punto de vista clínico.

## La acusación del “negocio de la prediabetes”

Es evidente la eficacia de los programas de intervención sobre el estilo de vida, pero el principal problema de los mismos suele ser la dificultad para el mantenimiento de los cambios recomendados, por lo que la prevención farmacológica podría ser una alternativa en algunos casos. Sin embargo, y a pesar de los datos demostrados por la intervención farmacológica sobre la reducción en la incidencia de diabetes, los fármacos hipoglucemiantes no tienen la indicación aprobada para su uso en estados prediabéticos.

Aún así, diversos autores opinan que la prediabetes es poco más o menos que un diagnóstico inventado con el objeto de medicalizar innecesariamente a la población [20]. Sin embargo, hay datos contundentes que, en mi opinión, rechazan este argumentario. Por un lado, solo la ADA [21] contempla claramente la posibilidad de utilizar metformina para la prevención de la DM2 en pacientes con un riesgo muy alto para desarrollar diabetes, como aquellos con ITG (nivel de evidencia A), GBA (nivel E), o una HbA_1c_ entre 5,7–6,4% (nivel E), especialmente si además presentan un IMC > 35 kg/m^2^, tienen menos de 60 años (eficacia mayor en este rango de edad demostrada en el estudio DPP) o son mujeres con diagnóstico previo de diabetes gestacional (nivel de evidencia A). Por otra parte, en un estudio recientemente publicado en *Diabetes Care*, se objetivó que el uso de la metformina en pacientes con prediabetes es menor del 1%, lo que demuestra que la farmacología está poco presente en la estrategia preventiva de la DM2 [22].

Para finalizar, después de esta fase de dudas sobre la realidad e importancia de la prediabetes, la investigación se está centrando no solo en su existencia sino incluso en los subtipos de prediabetes que podemos encontrar en la población [23]. Los distintos grupos metabólicos identificados se asocian con futuras complicaciones relacionadas con la prediabetes, la resistencia a la insulina, el riesgo futuro de DM2 y la mortalidad. Estos subfenotipos probablemente reflejan características patológicas clave que potencialmente subyacen a diferentes destinos de las complicaciones metabólicas, pero no tienen como objetivo clasificar a pacientes individuales en la práctica clínica; sin embargo, con un mayor desarrollo y validación, dichos enfoques podrían orientar las estrategias de prevención y tratamiento de las enfermedades cardiovasculares y renales, así como de la DM2. Así pues, tenemos que seguir atentos a las próximas noticias sobre esta interesante condición para saber de qué estamos hablando cuando hablamos de prediabetes.
